# Protective and restorative effects of the traditional Chinese medicine Jitai tablet against methamphetamine-induced dopaminergic neurotoxicity

**DOI:** 10.1186/s12906-018-2094-z

**Published:** 2018-02-23

**Authors:** Shasha Xu, Shaoang Tu, Jinlong Gao, Jia Liu, Zhirui Guo, Jinming Zhang, Xingdang Liu, Jianhui Liang, Yiyun Huang, Mei Han

**Affiliations:** 1 Key Laboratory of Radiopharmaceuticals, Ministry of Education, College of Chemistry, Beijing Normal University, 19 Xinjiekouwai Street, Haidian district, Beijing, China; 2 Chinese PLA General Hospital, Beijing, China; 3 Department of Nuclear Medicine, Huashan Hospital, Fudan University, Shanghai, China; 4 Department of Molecular and Cellular Pharmacology, Peking University School of Pharmaceutical Sciences, Beijing, 100191 China; 5 PET Center, Department of Radiology and Biomedical Imaging, Yale University School of Medicine, New Haven, USA

**Keywords:** Methamphetamine, Neurotoxicity, Jitai tablet, Dopamine transporter, Dopamine D2 receptor, Tyrosine hydroxylase

## Abstract

**Background:**

Methamphetamine (METH) is a psychostimulant with high abuse liability that affects the monoamine neurotransmitter systems, particularly the dopamine system. Currently there are no effective medications for the treatment of METH abuse to restore METH-induced dopaminergic dysfunction. The Jitai tablet (JTT), a commercial traditional Chinese medicinal preparation, has been shown to modulate the dopaminergic function both in heroin addicts and in morphine-dependent rats. The purpose of this study was to investigate, in a rodent model, whether JTT can protect against METH-induced neurotoxicity, and/or restore METH-damaged dopaminergic function.

**Methods:**

Immunohistochemical staining and/or autoradiography staining were used to detect tyrosine hydroxylase (TH) expression in the substantia nigra, and to examine the levels of dopamine transporter (DAT), dopamine D2 receptor (D2R) and TH levels in the striatum. Using a stereotyped behavior rating scale, we evaluated the inhibitory effect of JTT on METH-induced behavioral sensitization.

**Results:**

Repeated METH administration induced obvious stereotyped behavior and neurotoxicity on the dopaminergic system. Pre-treatment with JTT significantly attenuated METH-induced stereotyped responses, and interdicted METH-induced changes in the levels of DAT, D2R and TH expression. Treatment with JTT after METH administration restored DAT, D2R and TH expression to normal levels.

**Conclusions:**

Our results indicated that JTT protects against METH-induced neurotoxicity and restores the dopaminergic function, and thus might be a potential treatment for the dopaminergic deficits associated with METH abuse.

## Background

Methamphetamine (METH) abuse has recently become a significant public health concern worldwide. The amphetamine-type stimulants, of which METH accounts for the largest share, remains the second most commonly used class of illicit drugs worldwide, with approximately 35.7 million reported users in 2014, and increasing [1]. The rapidly escalating use of METH, especially the high frequency of METH use among young individuals aged 18–25 years, has caused significant health problems and a worldwide economic burden. On average, people seeking treatment for METH abuse are younger (average 25 years old) than those of traditional drugs such as opioids and cocaine, especially in Asia where there has been a substantial increase in the treatment for METH abuse. Hence, METH abuse is an issue that requires global attention and clinically significant treatment interventions.

The dopaminergic system plays an important role in drug abuse and neurotoxicity. Acute administration of METH acts on the dopamine transporter (DAT), resulting in increased release of dopamine (DA) [2], and leading to the stereotyped behavior [3, 4]. The massive release of dopamine leads to the oxidative stress through the generation of reactive oxygen species including superoxide radicals and dopamine quinones, and then cause the long-lasting neuronal damage [5]. The neurotoxicity on the dopaminergic system induced by repeated exposure to METH, shows up as a reduction in tyrosine hydroxylase (TH) activity, DA concentration and DAT levels, and loss of DA terminals and cell bodies [6]. Both postmortem and neuroimaging studies have documented reduced levels of DA, TH and DAT in the brains of chronic METH abusers [7–10]. In addition, PET imaging studies have reported that METH appears to induce long-lasting decreases in dopamine D2 receptors (D2R) [11] and persistent decreases of 20–30% in striatal DAT [12], and that this reduction can last as long as 3 years after METH withdrawal [8]. These neurobiological changes in the dopaminergic system are believed to contribute to the difficulties in abstinence treatment and the high relapse potential. Therefore, medications that target dopaminergic pathways are presumed to have potential for the treatment of METH abuse.

At present, few effective treatment options exist for METH use disorder besides psychosocial interventions [13]. Recent advances in the knowledge of the neurobiological mechanisms of METH abuse have promoted the development of pharmacologic treatments. The present pharmacotherapy interventions for the treatment of METH dependence or withdrawal are mainly antipsychotics, non-amphetamine stimulants, and antidepressants. The serotonin neurotransmission enhancer mirtazapine, the opioid receptor antagonist naltrexone and the DA and norepinephrine reuptake inhibitor bupropion appear to be promising. However, substantial evidence is still lacking on their efficacy in the treatment of METH abuse, especially in clinical trials [14]. In addition, none of these experimental pharmacotherapies have been shown to be effective in ameliorating METH-induced neuronal injury, which may be critical for a successful recovery. Therefore, there is still an urgent need for more effective agents specifically designed to protect or restore the dopaminergic system from METH-induced neurotoxicity.

Traditional Chinese medicine has been used for drug addiction treatment for more than 170 years and useful experiences have been accumulated with respect to patients’ detoxification [15]. Jitai tablet (JTT) is a commonly used traditional Chinese medicine that has been approved by the China Food and Drug Administration for opioid addiction treatment. The JTT prescription consists of 15 herbs (with 101 compounds tentatively identified previously) [16], including Papaveraceae Corydalis (10.2%), Solanaceae Daturametel (2.18%), Lamiaceae Salvia Miltiorrhizae (16.87%), Araliaceae Panaxginsen (2.18%), Apiaceae Angelica Sinensis (10.20%), Ranunculaceae Aconitum (2.18%), Myristicaceae Myristicacagayanensis (2.18%), Asteraceae Aucklandia (5.71%), Thymelaeaceaceae Aquilaria, (4.35%), Zingiberaceae Zingiber (2.18%), Lauraceae Cinnamomum (2.18%), Semen Persicae (10.20%), Pearl powder (13.47%). Previous studies from our laboratory have shown JTT to have both protective and restorative effects on the dopaminergic system in animal models of morphine addiction and 1-methyl-4-phenyl-1,2,3,6-tetrahydropyridine (MPTP) induced Parkinson’s disease [17, 18]. Furthermore, previous clinical single photon emission computed tomography (SPECT) imaging study reported that JTT appeared to stimulate the recovery of DAT in chronic heroin users [19]. Therefore, as both METH and opioids cause similar impairments of dopaminergic system function [20], we postulate that JTT may also be effective in the protection and/or restoration of the dopaminergic system impaired by repeated METH administration.

The aim of the present study was to evaluate the protective and restorative effects of JTT on METH-induced dopaminergic impairment. In addition, a stereotyped behavior test was conducted after each METH injection to determine whether pre-treatment with JTT could attenuate METH-induced behavioral performance.

## Methods

### Animals

Male Wistar rats (weighing 180–220 g) were caged in groups of five in a humidity- and temperature-controlled room, and kept under a 12 h: 12 h light/dark cycle, with free access to water and standard rodent food. Experimental procedures concerning animals were approved by the Ethics Committee of Beijing Normal University (BNU/EC/01/2011). All experimental procedures were performed in keeping with National Institutes of Health Guideline for the care and use of laboratory animals (NIH Publications No. 80–23, revised 1996).

### Jitai tablets

JTT (batch number: 050602) were kindly provided by the National Engineering Research Center for Traditional Chinese Medicine (Shanghai, China). JTT was dissolved in deionized water and administered intragastrically. The fingerprint of the JTT used in this study was presented in the supplement material of our previously published article [18].

### Treatment procedures

The rats were randomly divided into the following groups: control group, METH treatment group, JTT pre-treatment group (0.087 g/kg), and three JTT post-treatment groups with low (JTT-L, 0.029 g/kg), medium (JTT-M, 0.087 g/kg), and high dose (JTT-H, 0.290 g/kg) of JTT. All groups except for the control group were administered METH to establish acute METH neurotoxicity, via intraperitoneal injections twice daily at 4 h intervals at a dosage of 15 mg/kg for 2 d, according to a previously reported method [21], with slight modifications. The control group received equivoluminal 0.9% NaCl solution. In the JTT pre-treatment group, JTT at a dose of 0.087 g/kg was administered to the rats through oral gavage for 7 d before METH injections. After METH administrations, the rats in the JTT post-treatment groups were administered with JTT once daily at doses of 0.029, 0.087, and 0.290 g/kg for 10 d, while rats in the control and METH groups received the same amount of deionized water.

The post-treatment of JTT was scheduled to examine its therapeutic ability to interdict the METH-induced decreases in DAT, D2R and TH expression. To confirm whether there is a dose-dependent effect, we designed three doses of JTT in the post-treatment regimen with ratio 10: 3: 1 and the high dose was directly converted from the clinical dose, namely 0.290 g/kg [22, 23]. The pre-treatment of JTT was scheduled to investigate whether JTT could attenuate METH-induced stereotyped responses, and interdicted METH-induced changes in the levels of DAT, D2R and TH expression. To avoid unnecessary animal use in consideration of the international guidelines for care and use of laboratory animals, we set only one dosage for pre-treatment in our experiments.

### Ratings of stereotyped behavior

Stereotyped behavior test (*n* = 10 for each group) was conducted using the following scale [24]: (0) stationary, little or no movement; (1) active, occasional to frequent movement; (2) active with episodes of repetitive forward head searching; (3) continuous forward head searching; (4) frequent repetitive rearing, side-to-side weaving or turning; (5) episodes of rapid jerking side-to-side, circular or dorsoventral head movements. Stereotyped behaviors were scored by a rater unaware of the specific experimental conditions. After each METH injection, the rats in the control group, JTT pre-treatment group and METH group were placed at the center of the chamber for a 5-min acclimation period. Then, the stereotyped behaviors of each rat were scored every 5-min over the next 60 min by an observer blind to the treatments. The positive stereotyped behavior was defined if the score was above 2 points for more than 15 min.

### Brain tissue preparation

At the end of the treatment, the rats were sacrificed by decapitation. The brains were removed rapidly and stored at − 80 °C until further use. The frozen brains were cut into 18-μm coronal sections with a cryostat microtome at − 20 °C (CM1900; Leica, Germany) for immunohistochemistry and autoradiography experiments.

### Immunohistochemical staining

Analyses of DAT, D2R and TH immunoreactivity in the striatum and TH immunoreactivity in the substantia nigra (SN) were conducted on frozen brain sections (*n* = 5 for each group). The selected slices were prewashed in phosphate buffer solution (PBS) (0.01 mol/L, pH = 7.4) for 5 min × 3 times and then treated with PBS containing 0.2% Triton X-100 for 5 min, then with 0.3% H_2_O_2_ in PBS for 10 min and then washed in PBS for 5 min × 3 times. After that, the slices were treated with 10% normal goat serum for 10 min (or with normal donkey serum for TH), followed by incubation at 4 °C for 20 h with primary antibodies (1: 200 dilution of D2R antibody AB5084P and 1: 100 dilution of DAT antibody MAB369 from EMD Millipore Corporation, Billerica, MA, USA; 1: 10,000 dilution of TH antibody T1299 from Sigma-Aldrich Co., St. Louis, MO, USA). The slices were then washed with PBS and incubated at 37 °C for 60 min with secondary antibodies (anti-rabbit antibody PV-6001 and anti-rat antibody ZB-2307 from ZSGB-BIO, Beijing, China; anti-mouse antibody PK4002 from Vector Laboratories, Inc., Burlingame, CA, USA). After the second incubation, the slices were washed in PBS for 3 min × 5 times and incubated for 3 min in 100 μL of 3,3′-diaminobenzidine tertrahydrochloride for visualization. The slices for TH staining were incubated with the ABC reagent (VECTASTAIN ABC kit, ZSGB-BIO, Beijing, China) at 37 °C for 30 min before visualization. The slices were finally washed in distilled water. The sections were counted using a bright-field microscope (M165FC, Leica) and optical densities of DAT, D2R and TH were calculated by Image-Pro Plus software.

### Autoradiography experiments in vitro and in vivo

Autoradiography experiment (*n* = 5 for each group) was conducted to confirm the results from immunohistochemical staining experiments, and also to provide evidence for future clinical studies using nuclear molecular imaging technology.

An in vitro autoradiography method was used for the analysis of D2R levels using the radioligand [^125^I]IBZM, which was prepared according to procedures described previously [25]. For D2R autoradiography, the brain slices were washed at room temperature for 20 min in 50 mmol/L Tris buffer (pH 7.4, containing 120 mmol/L NaCl, 5 mmol/L KCl, 2 mmol/L CaCl_2_, 1 mmol/L MgCl_2_). The slices were then incubated for 60 min in the same buffer with 50 pM [^125^I]IBZM for D2R total binding. Nonspecific binding was determined in adjacent slices in the presence of 10 μM sulpiride (D2R antagonist, Sigma-Aldrich Co., St. Louis, MO, USA). After incubation, the slices were washed (1 min × 5 times) in ice-cold 50 mM Tris buffer (pH 7.4) and then rapidly dipped in deionized water and dried under a stream of cold, dry air. The labeled slices were exposed to super sensitive phosphor screen (PerkinElmer, USA) for 2 h. The exposed film was used for densitometry analysis.

In vivo autoradiography was conducted to analyze DAT levels. Five rats from each group were injected intravenously via the tail vein with 0.2 mCi [^99m^Tc]TRODAT-1, which was prepared according to the literature method described previously with minor modifications [26]. At 120 min post-injection, the rats were sacrificed by decapitation. The brains were removed and placed in liquid nitrogen for 1 min, and then stored at − 80 °C for 10 min. The frozen brains were cut into 18-μm coronal sections with a cryostat microtome at − 20 °C. These frozen slices were then exposed to super sensitive phosphor screen (PerkinElmer, USA) overnight. The exposed film was used for densitometry analysis.

The densitometry determinations were carried out with a Cyclone-Plus phosphor storage system (PerkinElmer, USA), and then analyzed with the Opti-Quant software (PerkinElmer, USA). Specific binding was calculated by subtracting the non-specific binding from that of the total binding. Results were presented as the ratio relative to the control group.

### Statistical analysis

The data were expressed as the means ± SD. Two-factor repeated measure analysis of variance (ANOVA) with time as a repeated measure (time × treatment) followed by LSD as post hoc test was used to analyze the data on stereotyped behaviors. For immunohistochemical staining and autoradiography results, one-way ANOVA was used, followed by Tukey’s HSD post hoc test. All of the statistical analyses were performed in SPSS 20.0, with *p* < 0.05 considered significant.

## Results

### Effect of JTT pre-treatment on METH-induced stereotyped behavior

**Fig. 1:**
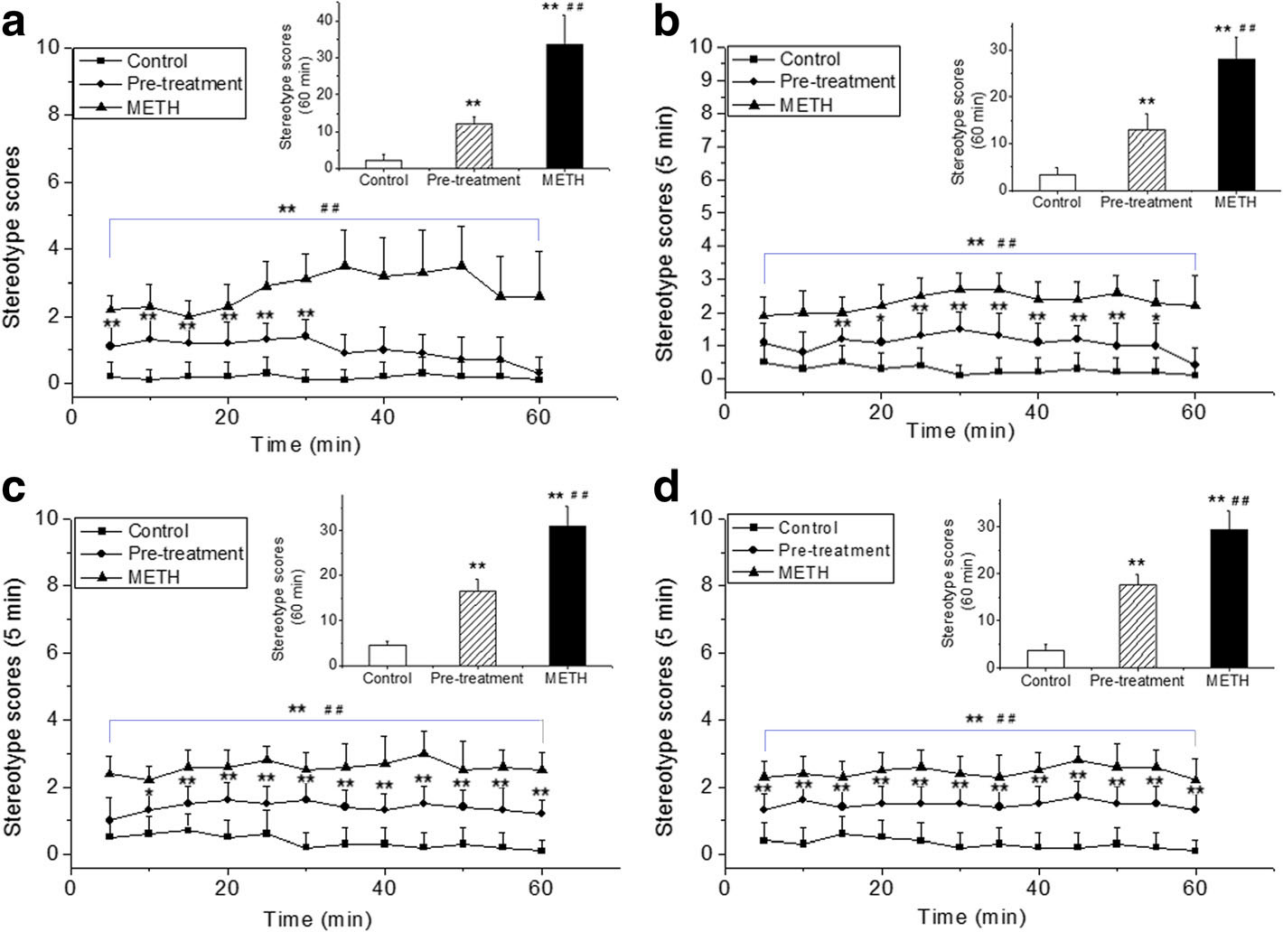
Effects of Jitai tablet (JTT) on stereotyped behavior after each methamphetamine (METH) injection. After the first (**a**), second (**b**), third (**c**) and fourth (**d**) injection of METH, rats in the METH group all exhibited obvious stereotyped behavior and the control group did not show any behavioral changes, whereas animals in the JTT pre-treatment group had attenuated stereotyped behavior in comparison with the METH group. Data are expressed as mean ± SD (*n* = 10). METH group or JTT pre-treatment group vs. control group: ** *p* < 0.001; METH group vs. JTT pre-treatment group: ^##^ *p* < 0.001

Stereotyped behavior rating scales were used to assess the behavioral effects of neurotoxicity induced by repeated METH administration. The positive stereotyped behavior was defined as a score of more than 2 points for more than 15 min. As shown in Fig. 1, the rats in the METH group exhibited obviously positive stereotyped behavior, as evidenced by the recorded stereotype scores in the METH group, which were > 2 points after each injection with METH. In contrast, animals in the JTT pre-treatment group exhibited moderate stereotyped behavior (stereotype scores, < 2 points during the whole assessment period).

The cumulative stereotype scores at 60 min were also analyzed. After each injection with METH,the rats showed significantly stereotyped behavior, as the total stereotype scores in 60 min in METH group or JTT pre-treatment were significantly higher than those in control group (*F*_*treatment*_ (2, 18) = 418.2, *p* < 0.001, *F*_*time*_ (3, 27) = 4.2, *p* = 0.014, *F*_*time × treatment*_ (6, 54) = 4.9, *p* = 0.01). Whereas JTT pre-treatment attenuated these behavior responses, as the stereotype scores decreased significantly in the JTT pre-treatment group compared to that in the METH group after each METH injection (*F* (1, 18) _*first injection*_ = 69.7, *F* (1, 18) _*second injection*_ = 62.6, *F* (1, 18) _*third injection*_ = 81.0, *F* (1, 18) _*forth injection*_ = 69.4, *p* < 0.001). These results indicate that JTT pre-treatment could effectively attenuate the METH-induced behavior response.

### Effects of JTT on DAT, D2R, and TH densities in the striatum, and TH densities in the SN of METH-dependent rats

#### JTT on DAT levels in the striatum

**Fig. 2:**
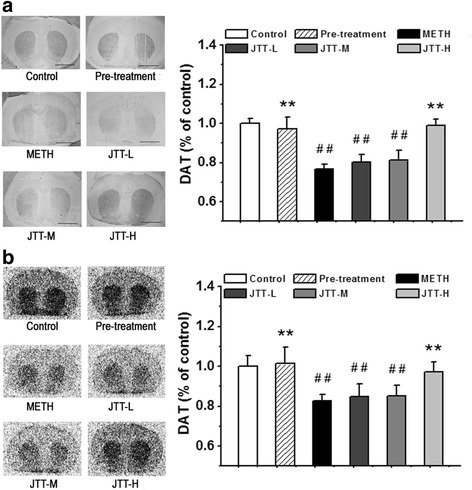
Effect of Jitai tablet (JTT) on dopamine transporter (DAT) levels in the striatum as assayed by immunohistochemical staining (**a**) (Bar = 5.00 mm) and autoradiography with [^99m^Tc]TRODAT-1 (**b**). Results of the two methods are accordant with each other. The remarkable reduction of DAT binding in the striatum induced by methamphetamine (METH) administration was significantly inhibited by JTT pre-treatment, or attenuated by post-treatment with high dose of JTT (JTT-H). Data are expressed as mean ± SD (*n* = 5). METH group vs. control group: ^#^ *p* < 0.05, ^##^ *p* < 0.01; JTT treatment group vs METH group: * *p* < 0.05, ** *p* < 0.01

DAT plays a key role in the reuptake of dopamine, and previous studies have documented that the chronic use of stimulants results in decreased DAT levels in the striatum [27]. Immunochemical staining and autoradiography with [^99m^Tc]TRODAT-1 were used to detect the effect of JTT pre- and post-treatment on DAT expressions in the striatum. As shown in Fig. 2a, the immunochemical staining results indicate that METH administration led to a significantly reduction in DAT levels to 76.6 ± 2.6% of that observed in the control group (*p* < 0.01). JTT pre-treatment completely inhibited this decrease and DAT densities were maintained at normal levels (97.2 ± 6.0% of the control levels), which were significantly higher than those in the METH group (*p* < 0.01). In addition, DAT levels in the group treated with high-dose JTT after METH administration were restored to normal levels (98.9 ± 3.4% of the control levels), which were also significantly higher than those in the METH group (*p* < 0.01).

Results from autoradiography (Fig. 2b) were consistent with those from immunohistochemical staining. The remarkable reduction in striatal DAT binding induced by METH administration (DAT binding at 82.7 ± 3.2% of the control levels) was completely blocked by JTT pretreatment (DAT binding at 101.4 ± 8.1% of the control levels, *p* < 0.01, compared with the METH group), or significantly attenuated by post-treatment with high dose of JTT (DAT binding at 97.3 ± 4.8% of the control levels for the JTT-H group, *p* < 0.01, compared with the METH group). These results indicate that JTT protect against METH-induced DAT impairments in the striatum of rats.

#### JTT on D2R expression in the striatum

**Fig. 3:**
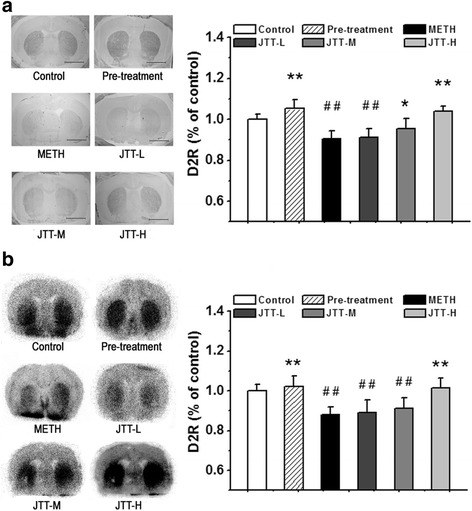
Effects of Jitai tablet (JTT) on dopamine D2 receptor (D2R) levels in the striatum by immunohistochemical staining (**a**) (Bar = 5.00 mm) and autoradiography with [^125^I]IBZM (**b**). The significant decrease of D2R expression in the striatum was inhibited by JTT pre-treatment, or significantly attenuated by post-treatment with high dose of JTT (JTT-H). Data are expressed as mean ± SD (n = 5). METH group vs. control group: ^#^ *p* < 0.05, ^##^ *p* < 0.01; JTT treatment group vs METH group: * *p* < 0.05, ** *p* < 0.01

The effects of JTT pre- and post-treatment on D2R expression in the striatum were determined by immunohistochemical staining (Fig. 3a) and autoradiography with [^125^I]IBZM (Fig. 3b). In the METH group, D2R density decreased to 90.6 ± 3.6% of the levels in the control group. Pre-treatment with JTT suppressed this decrease and maintained D2R at normal levels (105.2 ± 4.6% of the control group), which were significantly different from the levels observed in the METH group (*p* < 0.01). Additionally, post-treatment with JTT attenuated D2R reduction in a dose-dependent manner, with D2R at 95.3% ± 5.1 of the control level for the group post-treated with medium dose of JTT (JTT-M vs. METH, *p* < 0.05), and at 103.9 ± 2.4% of the control level for the group post-treated with high dose (JTT-H vs. METH, *p* < 0.01).

The above results were confirmed by autoradiography (Fig. 3b). The significant decrease of D2R binding (88.1 ± 3.7% of the control level) in the striatum was inhibited by JTT pre-treatment (102.3 ± 5.1% of the control level, *p* < 0.01), or post-treatment with high dose of JTT (D2R levels at 101.3 ± 5.2% of the control level, *p* < 0.01). These results suggest that JTT protect against METH-induced D2R loss in the rat striatum.

#### JTT on TH-positive neurons in the striatum and SN

**Fig. 4:**
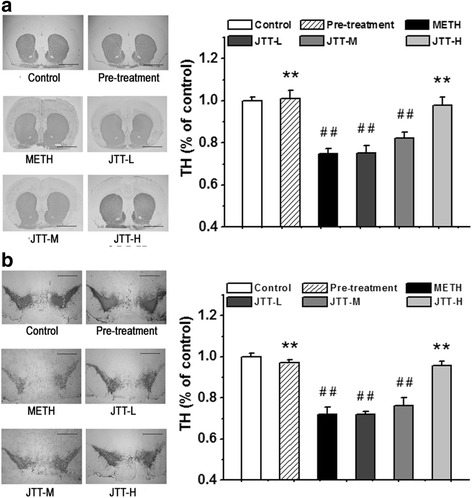
Effects of Jitai (JTT) on optical densities of tyrosine hydroxylase (TH) in the striatum (**a**) (Bar = 5.00 mm) and substantia nigra (SN) (**b**) (Bar = 2.00 mm). The loss of TH-positive neurons in the striatum and SN due to methamphetamine (METH) administration were suppressed by JTT pre-treatment, or reversed by post-treatment with a high dose of JTT (JTT-H). Data are expressed as mean ± SD (n = 5). METH group vs. control group: ^#^ *p* < 0.05, ^##^ *p* < 0.01; JTT treatment group vs METH group: * *p* < 0.05, ** *p* < 0.01

TH is a key enzyme in DA synthesis and generally used as a marker for dopaminergic neurons [28–31]. In this study, immunohistochemical staining was used to determine the concentration of TH-positive neurons and fibers in the striatum and SN. As shown in Fig. 4a, optical density analysis of TH-positive fibers revealed a significant loss of striatal DA terminals in the METH group as compared to that in the control group (74.7 ± 2.6% of the control levels, *p* < 0.01). Pre-treatment with JTT completely inhibited this loss and maintained TH expression at 101.2 ± 3.6% of the control group, which was significantly higher than that in the METH group (*p* < 0.01). Furthermore, significant elevations in the density of TH-positive fibers were observed in rats post-treated with high dose of JTT (TH level at 97.9 ± 3.9% of the control group, *p* < 0.01, in comparison with the METH group).

Figure 4b illustrates the optical density of TH-positive neurons in the SN. METH administration induced a marked loss of the concentration of TH positive neurons (71.9 ± 3.6% of the control group, *p* < 0.01). Rats pre-treated with JTT did not exhibit this reduction and maintained TH at 97.1 ± 1.6% of the control group level. Additionally, METH rats post-treated with a high dose of JTT exhibited significantly increased levels of TH expression (95.8 ± 1.9% of the control levels, in comparison with the METH group, *p* < 0.01). These results indicate that JTT protects against METH-induced loss of dopaminergic neurons in both the striatum and SN.

## Discussion

The present study is the first to evaluate the Jitai tablet as a novel treatment for METH-induced dopaminergic neurotoxicity. We found that pre-treatment with JTT (0.087 g/kg/day) effectively inhibited the stereotyped behavior and the changes in the levels of DAT, D2R and TH induced by repeated METH exposure. Post-treatment with high dose JTT (JTT-H, 0.290 g/kg/day) appeared to ameliorate METH-induced injury of dopamine neurons, as evidenced by the recovery of DAT and D2R levels in the striatum, and TH expression in the striatum and SN. Concentrations of DAT, D2R and TH in both the JTT pre-treatment group and JTT-H post-treatment group were no different from those in the control group. Our results suggest that JTT produced protective and restorative effect against METH-induced dopaminergic neurotoxicity.

Previous animal studies have shown that repeated administration of METH induces long-term dopamine terminal damage and neuronal body loss, as evidenced by the decrease in dopaminergic markers such as DAT, TH and D2R [32–34]. In the present study, we found that post-treatment with a high dose of JTT completely recovered the losses in striatal DAT, D2R, and TH induced by repeated METH administration. This was consistent with our previous studies showing that JTT effectively relieves morphine withdrawal symptoms by regulating the dopaminergic system, as well as our SPECT imaging studies, which reported that 6-month treatment with JTT significantly elevated striatal DAT levels in chronic heroin users [18, 35]. Therefore, JTT presents an excellent treatment possibility for METH-induced neurotoxicity through normalization of dopaminergic function and restoration of dopamine neurons in the striatum and SN.

Clinical study and epidemiological findings suggest that METH use is associated with an increased risk of developing Parkinson’s Disease (PD) [36–38]. Exposure to METH damages dopaminergic fibers in the striatum and their cell bodies in the SN, echoing the degeneration pattern observed in human patients with PD. In addition to the neurotoxicity of METH to the dopamine terminals in the striatum, our study also found a significant dopamine cell body loss in the SN, which was consistent with a previous animal study that reported a 20 to 25% loss of dopaminergic cells in the SN after repeated METH administration [39]. We found that pre-treatment with JTT at a dose of 0.087 g/kg completely prevented the loss of TH-positive cell bodies in the SN, while post-treatment with JTT at a dose of 0.290 g/kg effectively repaired the degeneration of dopamine neurons induced by METH administration. Our previous study showed that JTT has neuroprotective effects against impairment of TH-positive neurons in MPTP-induced PD model mice [17]. The treatment strategies for PD are generally aimed at halting the degeneration of nigral cells or restoring the function of remaining neurons. Thus, we postulate that the protective and repairing effects of JTT on METH-induced neuronal damages in the SN may play an important role in the prevention of METH users developing PD, as similar changes have been observed between METH users and PD patients, and METH users are at high risk of developing PD [37].

Multiple smaller doses of METH administration could induce acute behavioral toxicity (including stereotypic behavior), as well as neurotoxicity in dopaminergic neurons [5, 40]. Therefore, to investigate the anti-toxicity effects of JTT on acute METH rat model, we treated the rats with JTT for 7 d before METH injection, which is similar to that used in other studies [18, 41]. In our present study, pre-treatment with JTT significantly attenuated METH-induced stereotyped responses, and interdicted METH-induced changes in the levels of DAT, D2R and TH expression. This suggests that JTT might offer protection against dopaminergic neurotoxicity induced by repeated METH administration. Previous reports have demonstrated that the stereotyped behavior results from the increasing of synaptic levels of dopamine after acute METH administration [5]. In turn, the excessive dopamine levels lead to oxidative stress through the autoxidation and metabolism of synaptic DA and cause the long-lasting neuronal deficits [42]. Therefore, the attenuation effect of JTT on behavioral responses, may result from its antioxidant effect. Previous reviews have concluded that the numerous interacting mechanisms contributed to the damage by METH, including excitoxicity, oxidative stress, mitochondrial dysfunction, apoptosis and neuro-inflammation [43, 44]. Therefore, the complexity and diversity of METH-induced neurotoxicity may need the multiple targeting effects of traditional Chinese medicine JTT. Further studies are indispensable to investigate the detailed mechanisms of JTT’s neuroprotective effects.

The pharmacological mechanisms underlying JTT’s ability to regulate the behavior response and dopaminergic system in the METH model rats are likely due to multiple targeting as a result of the combined effects of its active ingredients. Many components in JTT have been identified, and the pharmacological effects for some of these bioactive components have been investigated in preclinical and clinical studies. For example, scopolamine, the active ingredient from the Solanaceae Daturametel, is a muscarinic cholinergic antagonist, and could enhance the dynamics of DA synthesis in the striatum through its antagonistic effects on muscarinic cholinergic neuronal activity [45]. Previous studies have showed that pretreatment with D2R antagonists could reduce or prevent the sensitized responses in rats repeatedly exposed to METH [46, 47]. Tetrahydroberineper and L-Tetrahydropalmatine (L-THP), the major ingredients from Solanaceae Daturametel, are DA receptor antagonists [48] and has been reported to inhibit METH-induced locomotor sensitization and to attenuate cocaine’s rewarding effects via its effect on DA receptor expression [49, 50]. In addition, L-THP has been reported to ameliorate heroin withdrawal syndrome, probably due to its ability to stimulate the synthesis and release of endogenous opioid peptides [48, 51]. Therefore, the attenuation effect of JTT on behavioral sensitization may result from the antagonism of its component, L-THP, on DA receptor. The mechanisms for METH-induced neurotoxicity are believed to include oxidative stress, mitochondrial dysfunction, N-methyl-D-aspartic acid receptor-mediated excitotoxicity and neuroinflammation [42]. The active ingredients of ginseng, which is also one of the herbs in JTT, have been shown both in animal models and cell cultures to mediate the protective effects on dopaminergic neurons through antioxidant, anti-inflammatory, anti-apoptotic and immunostimulant activities [52]. Ginseng has also been reported to inhibit cocaine-induced behavioral sensitization and dopamine release [53]. As there are multiple components in the JTT prescription, it is difficult to pinpoint the clear mechanisms for its modulation of the dopaminergic system. The mechanism underlying the protective and restorative effects of JTT on the METH-induced dopaminergic changes is likely to derive from the combined effects of its active ingredients. Its exact nature remains to be elucidated in further studies.

## Conclusions

In conclusion, this animal study was the first to evaluate the effects of the Chinese medicine Jitai tablet on dopaminergic system impairments inflicted by repeated METH administration. Evidence from the present study appears to indicate that JTT may inhibit the neurotoxicity induced by repeated METH exposure, as evidenced by the significant attenuation of METH-induced stereotyped behavior, as well as the inhibition of reductions in DAT, D2R and TH expression levels with JTT pre-treatment. In addition, JTT appears to afford restorative effects on the METH-induced dopaminergic damages, as evidenced by the significant recovery of DAT, D2R levels and TH activity in the striatum and the SN. Taken together, results from our present study document that JTT may be considered a promising therapeutic agent for METH use disorder. Further studies are warranted to examine the therapeutic efficacy of JTT in patients with METH use disorder.

## Abbreviations

ANOVA: Analysis of Variance
D2R: Dopamine D2 Receptor
DA: Dopamine
DAT: Dopamine Transporter
JTT: Jitai Tablet
L-THP: L-Tetrahydropalmatine
METH: Methamphetamine
MPTP: 1-methyl-4-phenyl-1,2,3,6-tetrahydropyridine
PBS: Phosphate Buffer Solution
PD: Parkinson’s Disease
SN: Substantia Nigra
SPECT: Single Photon Emission Computed Tomography
TH: Tyrosine Hydroxylase
